# GnRH receptor activation competes at a low level with growth signaling in stably transfected human breast cell lines

**DOI:** 10.1186/1471-2407-11-476

**Published:** 2011-11-03

**Authors:** Kevin Morgan, Colette Meyer, Nicola Miller, Andrew H Sims, Ilgin Cagnan, Dana Faratian, David J Harrison, Robert P Millar, Simon P Langdon

**Affiliations:** 1 Medical Research Council Human Reproductive Sciences Unit, The Queen's Medical Research Institute, Little France Crescent, Old Dalkeith Road, Edinburgh EH16 4TJ, UK; 2 Breakthrough Research Unit and Division of Pathology, Institute of Genetics and Molecular Medicine, University of Edinburgh, Crewe Road, Edinburgh, EH4 2XU, UK

## Abstract

**Background:**

Gonadotrophin releasing hormone (GnRH) analogs lower estrogen levels in pre-menopausal breast cancer patients. GnRH receptor (GnRH-R) activation also directly inhibits the growth of certain cells. The applicability of GnRH anti-proliferation to breast cancer was therefore analyzed.

**Methods:**

GnRH-R expression in 298 primary breast cancer samples was measured by quantitative immunofluorescence. Levels of functional GnRH-R in breast-derived cell lines were assessed using ^125^I-ligand binding and stimulation of ^3^H-inositol phosphate production. Elevated levels of GnRH-R were stably expressed in cells by transfection. Effects of receptor activation on *in vitro *cell growth were investigated in comparison with IGF-I and EGF receptor inhibition, and correlated with intracellular signaling using western blotting.

**Results:**

GnRH-R immunoscoring was highest in hormone receptor (triple) negative and grade 3 breast tumors. However prior to transfection, functional endogenous GnRH-R were undetectable in four commonly studied breast cancer cell lines (MCF-7, ZR-75-1, T47D and MDA-MB-231). After transfection with GnRH-R, high levels of cell surface GnRH-R were detected in SVCT and MDA-MB-231 clones while low-moderate levels of GnRH-R occurred in MCF-7 clones and ZR-75-1 clones. MCF-7 sub-clones with high levels of GnRH-R were isolated following hygromycin phosphotransferase transfection. High level cell surface GnRH-R enabled induction of high levels of ^3^H-inositol phosphate and modest growth-inhibition in SVCT cells. In contrast, growth of MCF-7, ZR-75-1 or MDA-MB-231 clones was unaffected by GnRH-R activation. Cell growth was inhibited by IGF-I or EGF receptor inhibitors. IGF-I receptor inhibitor lowered levels of p-ERK1/2 in MCF-7 clones. Washout of IGF-I receptor inhibitor resulted in transient hyper-elevation of p-ERK1/2, but co-addition of GnRH-R agonist did not alter the dynamics of ERK1/2 re-phosphorylation.

**Conclusions:**

Breast cancers exhibit a range of GnRH-R immunostaining, with higher levels of expression found in triple-negative and grade 3 cancers. However, functional cell surface receptors are rare in cultured cells. Intense GnRH-R signaling in transfected breast cancer cells did not markedly inhibit growth, in contrast to transfected HEK 293 cells indicating the importance of intracellular context. GnRH-R signaling could not counteract IGF-I receptor-tyrosine kinase addiction in MCF-7 cells. These results suggest that combinatorial strategies with growth factor inhibitors will be needed to enhance GnRH anti-proliferative effects in breast cancer

## Background

Endocrine suppression using gonadotropin releasing hormone (GnRH) analogs such as goserelin (a super-agonist) is commonly used for the treatment of pre-menopausal estrogen-responsive breast cancer because it lowers plasma levels of estrogen by inhibiting secretion of luteinizing hormone and follicle stimulating hormone from the pituitary gland [1,2] and thereby slows estrogen-driven tumor growth.

It has been speculated since a proportion of cancer cells express GnRH receptor, that activation or inhibition of GnRH receptor signaling may directly affect cell growth [3-5].

This could have therapeutic value in both ER-positive and ER-negative tumors if the GnRH-sensitive population could be identified. A range of *in vitro *and animal model studies have explored this phenomenon [5-10]. The cellular response to GnRH receptor activation is complex. Cell-type specific features influencing GnRH receptor signaling and cell growth-inhibition have been described in cell lines stably expressing elevated levels of the GnRH receptor [8-10]. So far, the ability of GnRH agonist to inhibit cell growth appears to correlate with the level of GnRH receptor expression at the cell surface and with the magnitude of inositol phosphate production elicited by receptor activation [8,9]. GnRH receptor activation coupled to Gα_q/11_-Gβγ proteins leads to elevation of intracellular Ca^2+ ^levels, altered cytoskeletal function and changes in protein kinase activity, including protein kinase C (PKC), mitogen activated serine/threonine kinases (MAPkinases, MAPK) and stress-activated kinases Cell-type specific effects of GnRH receptor activation on levels of phosphorylated-ERK1/2 (p-ERK1/2) have been observed [8,9] which probably reflect the complexity of protein scaffolds interacting with and influencing MAPK. Effects of GnRH receptor signaling on transcription factor activity and gene expression downstream from MAPK are also likely.

Previous studies have shown that the growth of some human breast cancer cells (MCF-7, MDA-MB-435 and -231) can be inhibited when GnRH receptor is targeted [6,7]. How this effect is achieved is only partially understood [4,10], but it may be more widely applicable to the regulation of breast cell growth.

Breast cancer is a highly heterogeneous disease arising through the accumulation of mutations in different cell types [11,12]. Individual cases can be characterized in increasing detail using microarray technology and complementary genomic data [13-21]. Consequently, a variety of alternative drug therapies are currently employed to treat breast cancer but new treatments aimed at 'personalized medicine' still need to be developed. Various inter- and intra-cellular signaling pathways driving cancer cell proliferation, involving steroid hormone receptors (estrogen receptor) and growth factor- or growth-factor-like receptors (the EGF receptor family and insulin-like growth factor receptor, IGF-IR), are targets for the development of new drugs [22-27]. How GnRH receptor signaling interacts with these pathways is an emergent area of study. Recent studies have suggested that breast cancers which possess low or zero levels of receptors for estrogen receptor, progesterone receptor and HER2 (triple negative cancers) have higher levels of GnRH receptor expression [5,7].

We analyzed GnRH receptor in 298 primary breast cancer tissue samples by quantitative immunofluorescence and screened breast cell lines for functional GnRH receptor. Several well characterized human breast cell lines known to possess different phenotypes and different oncogenic mutations expressing elevated levels of GnRH receptor were isolated following cDNA transfection. The effects of receptor activation on cell growth and intracellular signaling were studied in order to determine whether cell phenotype influences the response to GnRH activation and seek strategies to develop the use of GnRH receptor as a cancer therapeutic target.

## Methods

Most reagents were purchased from Sigma UK, including D-Trp^6^GnRH-I (D-Trp^6^-LHRH, Triptorelin). Antibodies for ERK-1/2 and phosphorylated-ERK1/2 were purchased from Cell Signaling Technology, UK and for β-actin, from Sigma, UK. Secondary antibodies conjugated to alkaline phosphatase were from Sigma, UK. Insulin like growth factor receptor-I (IGF-IR) inhibitor II, EGFR/ErbB2 inhibitor and phosphatidylinositol-4,5-bisphosphate 3-kinase γ (PI3Kγ) inhibitor were purchased from Calbiochem, UK. SVCT cells [28] were purchased from ECACC, UK. MCF-7, MDA-MB-231, ZR-75-1, and T47D cells were from American Type Culture Collection (LGC, UK). The GnRH receptor stably transfected HEK293_[SCL60] _and prostate WPE-1-NB26-8 cell lines described elsewhere [8,9] together with HEK293 cells were used as controls for comparison. These transfected models have previously been shown to demonstrate growth responses to triptorelin [8,9].

### Tissue microarray

Three tissue microarrays (TMAs) were constructed with triplicate samples from 298 primary breast carcinomas as previously described [29]. The primary tissue was collected after surgical breast resection between 1999 and 2002 at the Edinburgh Breast Unit, Western General Hospital, Edinburgh [29]. The study was approved by the Lothian Research Ethics Committee (08/S1101/41). No informed consent (written or verbal) was obtained for use of retrospective tissue samples from the patients within this study, most of whom were deceased, since this was not deemed necessary by the Ethics Committee, who waived the need for consent. Paraffin embedded sections were prepared from the TMAs (3 μm thick) using a microtome and then mounted onto slides. NCL-GnRHR (A9E4) Leica Microsystems antibody (Novocastra Laboratories, UK) was used to detect the level of endogenous GnRH receptor immune-staining across primary breast tumours by quantitative immuno-fluorescence (using AQUAnalysis software (HistoRx Ltd., USA), as previously described [30]. Data were normalized by mean-centering to reduce systematic variation between the three TMAs.

### Cell culture, transfection and clone isolation

Cells were cultured in Dulbecco's modified Eagle's medium (DMEM) with 10% fetal bovine serum. Medium for SVCT cells was supplemented with recombinant human insulin and hydrocortisone as specified by the suppliers (ECACC, UK). HEK293_[SCL60] _and WPE-1-NB26-8 cells were cultured as described elsewhere [9]. Cells were transfected with a plasmid construct, pcDNA3.1(+) (neo) (Invitrogen, UK) containing a rat GnRH receptor cDNA insert, using Fugene 6 (Roche, UK) in Optimem-I (GIBCO, Invitrogen, UK). Cell clones growing in 6 cm dishes were picked using trypsinization in cloning cylinders (Sigma, UK) and sequentially expanded in multiwell plates and flasks prior to characterization. Sub-clones were generated by re-transfecting an individual clone with a 2.334 kb SV40 promoter-hygromycin phosphotransferase cDNA fragment excised from pcDNA3.1(-) (hygro) plasmid (Invitrogen, UK) using PvuII (Promega, UK) and purified following agarose gel electrophoresis.

### GnRH binding assay

Levels of GnRH receptor at the cell surface were measured as described elsewhere, using ^125^I-labeled His^5^D-Tyr^6^GnRH-I as a radiotracer [8,9]. Cells were grown in 12 or 24 well plastic culture plates. The number of cells per well was determined on the day of assay using a hemocytometer to count trypsinized samples from wells prepared in parallel. For accurate determination of relative levels of GnRH receptor expression between different cell clones, binding assays were performed over a range of cell confluencies and the results adjusted for the number of cells per well. Non-specific binding was determined using empty wells and by the addition of 1 micromolar unlabeled mammalian GnRH-I (Sigma, UK) to displace specific binding of tracer from cells. Assays were performed in triplicate and were repeated on separate occasions to determine accuracy of measurement.

### In vitro cell growth assay

Cells were seeded into 12 well plates and growth was monitored using the sulforhodamine B (SRB) staining assay described previously [8,9]. Two milliliters culture medium per well was sufficient to sustain cell growth over all time courses investigated. Cells were treated with a dose range of Triptorelin or vehicle (20% propylene glycol, Sigma, UK). Similar experiments employing IGF-IR, EGFR/ErbB2 and PI3K inhibitors were performed. Assay measurements were performed in triplicate and were repeated on separate occasions. At each time point, cells were fixed by adding 1 ml 25% trichloroacetic acid to each well, stored at 4°C for 1 h before gently washing and drying plates. Fixed cells were stained with 0.4% SRB in 1% acetic acid, washed, dried and dissolved in 1 ml 0.1 M Tris pH 10. Absorbance measurements at 540 nm correlated with the number of cells per well.

### Inositol phosphate assay

Production of ^3^H-inositol phosphates was measured in cells grown in 12 or 24 well plates as described previously [8,9]. Results were standardized according to the number of cells per well on the day of assay, determined using spare wells prepared in parallel. Single-dose or dose-response experiments were performed in triplicate and on separate occasions. Cells were allowed to reach 50-70% confluence before overnight incubation in serum-free, inositol-free DMEM containing 1 uCi/ml ^3^H myo inositol. Medium was replaced with 1 ml/well HEPES + DMEM containing 0.1% BSA and 10 mM LiCl and plates incubated at 37°C for 30 min. This medium was then replaced with fresh medium containing vehicle or treatment and incubated at 37°C for 1 h. Medium was removed and cells were fixed with 1 ml/well 0.1 M formic acid and incubated at 4°C for 30 min. ^3^H-inositol phosphates were purified from the supernatant using Dowex ion exchange chromatography. The final eluate was measured using a scintillation counter.

### Western blotting

Cells were grown in six-well plastic culture plates until 50-70% confluent. Some samples were washed twice with phosphate buffered saline prior to incubation in serum-free medium overnight. Cells were treated with 100 nM Triptorelin or vehicle for specific time periods prior to lysis and harvesting. Samples were processed for western blotting as described previously using NP40 lysis buffer at at 4°C [8,9]. For quantitative data, time points were measured in triplicate. Blots were imaged by a Typhoon phosphor-imager (GE Healthcare, UK) using enhanced chemi-fluorescence detection and analyzed using ImageQuant software (GE Healthcare, UK).

### Inverse PCR analysis of DNA integration sites

Genomic DNA was prepared from MCF-7-30 cell sub-clones stably transfected with SV40 promoter-hygromycin resistance DNA fragment (hygroR). Aliquots of genomic DNA were digested with a single restriction endonuclease (Promega, UK) which cuts at only one site within the hygroR DNA fragment (either AvrII, PvuI, SacII or ScaI) and relegated to form circular DNA containing flanking DNA from the genomic integration site using T4 DNA ligase. Pairs of polymerase chain reaction (PCR) primers targeting the hygroR DNA, flanking the cut-religation site were used to amplify DNA adjacent to the hygroR integration site by walking away from the hygroR sequence. Purified PCR products were cloned into pcr4 sequencing vector (Invitrogen, UK) and subjected to automated DNA sequence determination.

### Graphical and Statistical analyses

Immuno-fluorescence data were analyzed by one-way ANOVA using Minitab version 16 (Minitab Inc., USA). Prism software (GraphPad, USA) was used to prepare graphs and to calculate EC_50 _and IC_50 _values. Western blots were quantified using ImageQuant software (GE Healthcare). Quantitative data were analyzed using on-line tools for T-test, http://easycalculation.com/statistics/standard-deviation.php and http://www.quantitativeskills.com/sisa/.

## Results

### GnRH receptor immuno-staining is highly variable across primary breast tumors but functional endogenous receptor is not detectable in breast cell lines

**Figure 1:**
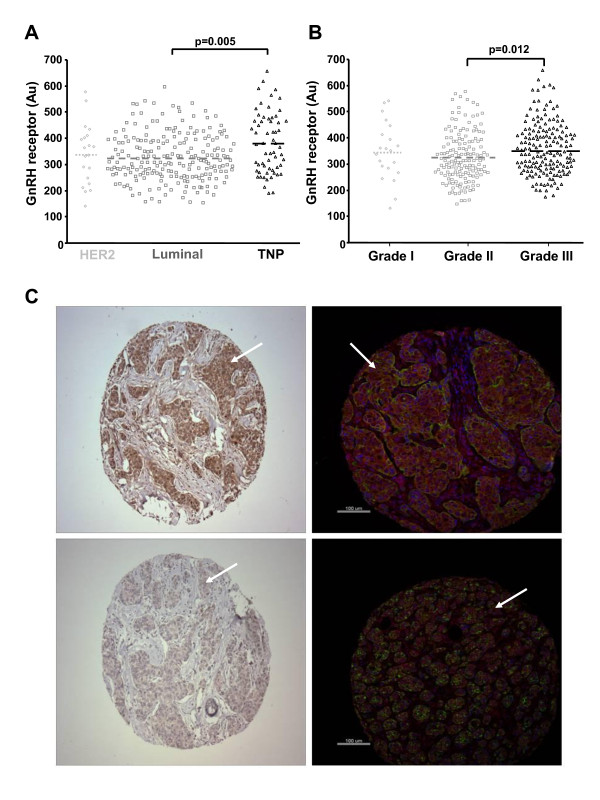
**GnRH receptor is expressed across a wide range in breast cancer and is highest in triple negative tumours when measured by immunostaining**. Association of GnRH receptor expression and (A) Cancer type and (B) Cancer grade. Quantitative immunofluorescence (AQUA) was used to measure GnRH receptor. One way ANOVA was used to test for significant differences between subtypes, the mean for each group is shown with a dashed line. C. Representative examples of high GnRH receptor expression (top images) and low expression (bottom images). Left hand images are immunohistochemical images of tissue microarray (TMA) cores of individual breast cancer with brown staining corresponding to GnRH receptor expression and blue to haematoxylin staining. Right hand images are immunofluorescence images of TMA cores, with red staining corresponding to GnRH receptor expression, blue (DAPI) staining indicating cell nuclei and green staining detecting cytokeration (ie carcinoma cell) staining. White arrows indicate areas of positive expression.

Tissue microarrays of 298 primary breast carcinomas from two cohorts of patients were examined by quantitative immunofluorescence (AQUA, HistoRx) for expression of GnRH receptor. The tumors were classified into three groups, triple negative phenotype (TNP, lacking ER, PR and HER2), HER2 positive or luminal [29]. There was a large dynamic range in the level of GnRH receptor staining (Figure 1) and the level was significantly higher in the TNP than luminal tumors (p = 0.005). GnRH receptor staining was also higher in grade 3 tumors compared to grade 2 tumors (p = 0.021).

**Figure 2:**
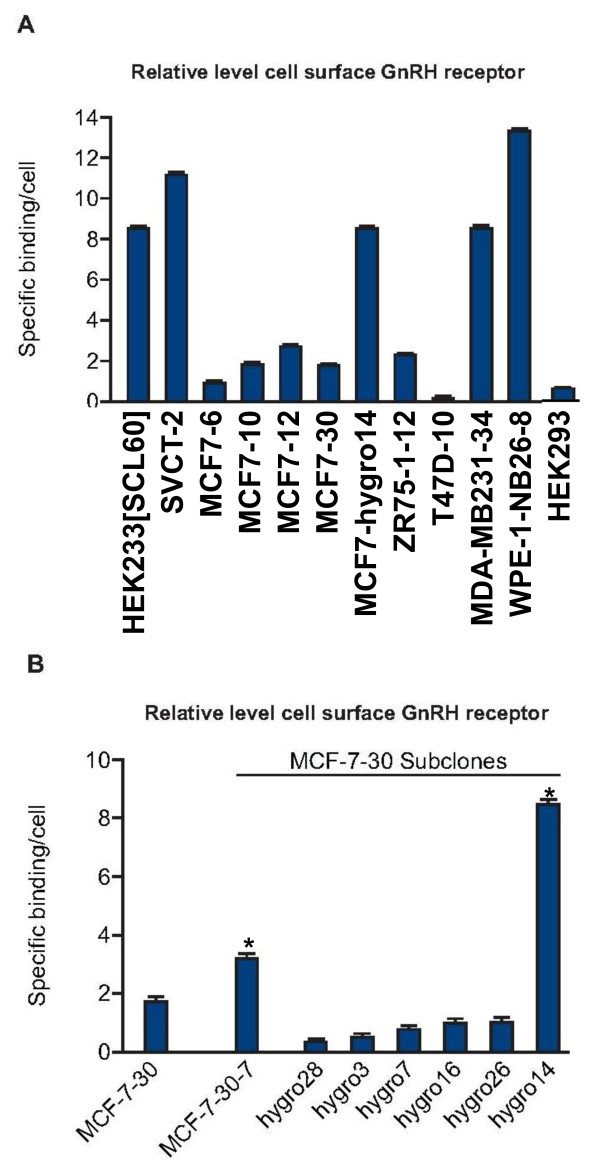
**Stably transfected breast cell lines can be generated with functional GnRH receptor**. Relative levels of GnRH at the cell surface detected by ligand binding assay in human cell lines stably transfected with rat GnRH receptor cDNA expression construct A. Subclones of MCF-7 clone 30 expressing modified levels of GnRH receptor at the cell surface were isolated. HEK293 and T47-D10 cells demonstrated background levels of binding. All other cell lines shown demonstrated significantly (p < 0.05 ANOVA) higher levels of specific binding. B. MCF-7-30-7 was subcloned from MCF-7-30 and then transfected with a PvuII SV40-hygromycin resistance gene fragment. Clones resistant to G418 and hygromycin were screened for altered GnRH receptor expression. GnRH receptor levels were elevated in clone MCF-7-30-hygro14, similar to levels in HEK293_[SCL60]_. * p < 0.05 (ANOVA followed by Dunnett's test) indicates significantly higher in MCF-7-30-7 and MCF-7-30-hygro14 relative to MCF-7-30 binding.

**Figure 3:**
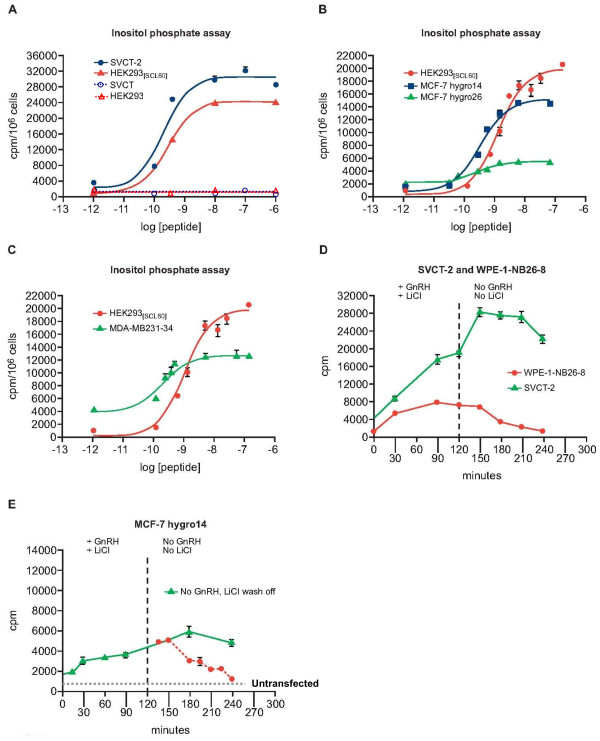
**Dynamics of GnRH receptor activation following treatment with Triptorelin**. Treatment of stably transfected cells with GnRH elicited high levels of ^3^H- inositol phosphate (IP) production (A-C). Removal of GnRH and LiCl revealed the dynamics of ^3^H- inositol phosphate turnover in different cell types (D,E). The decrease in levels of ^3^H- inositol phosphates was slower in SVCT-2 cells. Statistically different values (p < 0.05 ANOVA followed by Dunnett's test) compared to control values were as follows; for A, All values shown for SVCT-2 and HEK293_[SCL60] _cells > -10 log [peptide]; for B, all values shown > -10 log [peptide] for all 3 cell lines; for C, all values shown > -10 log [peptide] for both cell lines; for D, all values shown for SVCT-2 and all values up to 180 min for WPE-1-NB26-8; for E, all values from 30 min to 180 min.

Initial assessment of an immortalized human breast epithelial cell line (SVCT) and four human breast cancer cell lines (MCF-7, ZR-75-1, T47D and MDA-MB-231) indicated that these models did not possess detectable levels of endogenous GnRH receptor at the cell surface when analysed using a binding assay employing a ^125^I-labelled GnRH analog (His^5^-D-Tyr^6^-GnRH-I). The cells did not accumulate ^3^H-inositol phosphates following treatment with Triptorelin (Figures 2 and 3).

### Stably transfected breast cell lines can be generated with functional GnRH receptor

To model GnRH receptor positive breast cancer, the above-mentioned cell lines were transfected with a GnRH receptor cDNA expression construct in pcDNA3.1(+) neo and cells resistant to G418 were cloned. At least thirty G418-resistant clones derived from each cell line were screened for expression of GnRH receptor using the binding assay and classified according to relative level of receptor detectable at the cell surface. Relative levels of specific binding exhibited by representative clones are depicted in Figure 2A. One SVCT clone (SVCT-2) expressed high levels of GnRH receptor at the cell surface. Approximately 50% of transfected MCF-7 clones exhibited moderate levels of specific GnRH binding (clones MCF7-6, -10, -12 and -30 in Figure 2A). A proportion of transfected ZR-75-1 cell clones also expressed moderately high levels of specific GnRH binding (see clone ZR-75-1-12 in Figure 2A). One out of 30 transfected MDA-MB-231clones expressed high levels of GnRH receptor, but no transfected T47D clones exhibited GnRH binding (Figure 2A). MCF-7hygro 14 cells were sub-cloned from MCF-7-30 cells by re-cloning (to generate MCF-7-30-7) followed by transfection with a promoter-hygromycin resistance gene fragment (hygro) and followed again by further sub-cloning. Of these sub-clones, MCF-7hygro14 possessed the highest levels of cell surface GnRH receptor (see Figure 2B for examples). Analysis of the integration site of the hygromycin resistance gene, using restriction endonuclease excision, DNA circularization, inverse PCR-cloning and DNA sequencing, indicated insertion immediately 5' to the CMV promoter driving transcription of the rat GnRH receptor cDNA in MCF-7hygro14. In all other MCF-7hygro clones investigated, the hygro gene was inserted adjacent to the 3' flank of the rat GnRH receptor cDNA (data not shown).

Levels of cell surface GnRH receptor in SVCT-2, MCF-7hygro14 and MDA-MB231-34 were similar to levels in stably transfected HEK293_[SCL60] _cells and prostate WPE-1-NB26-8 cells described elsewhere [8,9] (Figure 2A).

The presence of functional GnRH receptor in these clones was confirmed by measuring production of ^3^H-inositol phosphates following addition of Triptorelin. SVCT-2, MCF-7hygro14 and MDA-MB-231 cells expressing rat GnRH receptor generated elevated levels of ^3^H- inositol phosphates following GnRH receptor activation which correlated with receptor expression level (Figure 3A-C). MDA-MB-231-34 cells exhibited elevated basal phospholipase C activity (Figure 3C). The dynamics of inositol phosphate accumulation following GnRH receptor activation were similar in the different cell lines but differences in turnover following removal of inositol phosphatase inhibition (LiCl wash off) occurred according to the cell line (Figure 3D and 3E). The decrease in levels of ^3^H- inositol phosphates was slower in SVCT-2 cells.

### The GnRH super-agonist Triptorelin had little or no effect on growth compared to inhibitors of IGFR-1 or EGFR

**Figure 4:**
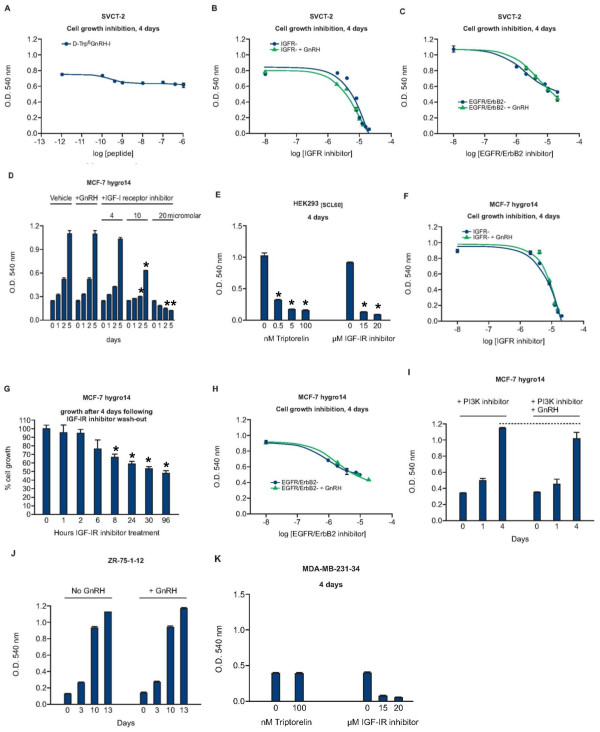
**The effect of Triptorelin on growth of cells stably transfected with GnRH**. A. Growth of SVCT-2 after 4 days was marginally inhibited (10-18%) by treatment with Triptorelin. However, cell growth was effectively inhibited by IGF-IR inhibitor II and co-treatment with Triptorelin exerted a small additive effect (B). EGFR/ErbB2 inhibitor reduced SVCT-2 cell growth, but co-treatment with Triptorelin had no effect (C). Growth of MCF-7-30-7hygro14 cells was not affected by treatment with 100 nM Triptorelin (D), unlike HEK293_[SCL60] _cells (E) after 4 days. Growth and survival were inhibited by IGF-IR inhibitor II but co-treatment with Triptorelin had no effect (F). Transient exposure to 15 μM IGF-IR inhibitor II for up to 2 hours resulted in less than 10% growth-inhibition after 4 days, longer exposures resulted in more extensive growth-inhibition (G). Growth of MCF-7-30-7hygro14 cells was inhibited by EGFR/ErbB2 inhibitor but not affected by treatment with 7 μM PI3Kγ inhibitor and co-treatment with 100 nM Triptorelin exerted no significant growth-inhibition (H and I). Growth of ZR75-1-12 (J) and MDA-MB-231-34 (K) was unaffected by treatment with 100 nM Triptorelin. * p < 0.05 (ANOVA followed by Dunnett's test).

The effects of Triptorelin on cell growth were investigated for a number of the stably transfected clones. Growth of SVCT-2 was modestly inhibited by treatment with Triptorelin (~10-18% inhibition relative to vehicle treated cells after 4 days, Figure 4A), with an IC_50 _of approximately 0.3 nM. In contrast, application of IGF-IR inhibitor II resulted in complete growth inhibition accompanied by cell death, with an IC_50 _of ~11 μM). Co-treatment with 100 nM Triptorelin had a small additive growth-inhibitory effect, shifting the IGF-IR inhibitor growth-inhibition dose-response curve slightly to the left (Figure 4B), reducing the apparent IC_50 _to ~9 μM. Treatment of SVCT-2 cells with EGFR/ErbB2 inhibitor resulted in a 50% growth-inhibition after 4 days, with IC_50 _of ~ 2 μM and co-treatment with 100 nM Triptorelin did not significantly affect growth in these experiments (Figure 4C).

Growth of MCF-7hygro14 was not affected by GnRH receptor activation, in contrast to the effect on HEK293_[SCL60] _cells (Figure 4D and 4E). Treatment of MCF-7hygro14 with IGF-IR inhibitor II resulted in growth-inhibition and cell death (IC_50 _~17 μM) and co-treatment with 100 nM Triptorelin had no significant effect (Figure 4F). Time-course experiments indicated that growth-inhibition could be reduced following washout of IGF-IR inhibitor II using phosphate buffered saline followed by replacement with normal culture medium. Growth-inhibition could be reduced to less than 10% over 4 days if the inhibitor was removed after a 2 hour exposure. Treatments for 6 hours or more resulted in growth-inhibition of more than 20% (Figure 4G). Treatment of MCF-7hygro14 cells with EGFR/ErbB2 inhibitor resulted in a 50% growth inhibition after 4 days, with IC_50 _of ~ 5 μM and co-treatment with 100 nM Triptorelin did not significantly affect growth in these experiments (Figure 4H). Dose-response studies using a PI3K inhibitor (ranging from 5 nM to 7 μM) indicated that the maximum dose did not affect growth over 4 days and co-treatment with 100 nM Triptorelin did not significantly alter this result (Figure 4I).

Growth of ZR-75-1-12 (slow growing) and MDA-MB-231-34 was also not affected by treatment with Triptorelin (Figure 4J and 4K).

### The levels of p-ERK1/2 were influenced by integration of signaling from multiple cell surface receptors which blocked responses to activated GnRH receptor

**Figure 5:**
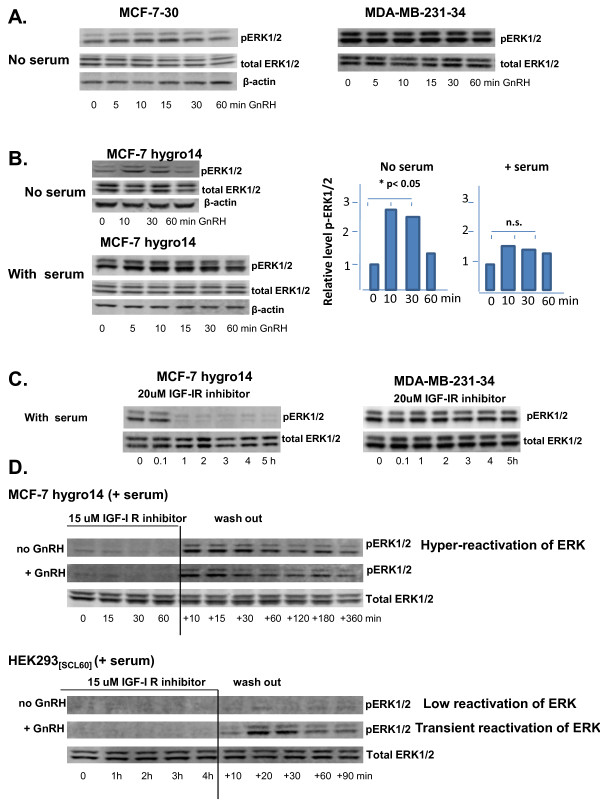
**The level of p-ERK1/2 is influenced by integration of signaling from multiple cell surface receptors, blocking the response to activated GnRH receptor**. A. Triptorelin did not affect levels of p-ERK1/2 in serum-starved MCF-7-30 or MDA-MB-231-34 cells. B. Treatment of stably transfected cells with 100 nM Triptorelin transiently elevated levels of phosphorylated ERK1/2 (p-ERK1/2) in serum-starved MCF-7-30-7hygro14 cells but not in the presence of serum. Bar graphs indicate effect of no serum vs with serum on ERK response in MCF7hygro14, statistically significant for no serum, p < 0.05. C. Treatment with IGF-IR inhibitor resulted in rapid and sustained de-phosphorylation of ERK1/2 in MCF-7-30-7hygro14 cells but not in MDA-MB-231-34 cells. D. Rapid re-phosphorylation of ERK1/2 occurred in MCF-7-30-7hygro14 cells when IGF-I receptor inhibitor was washed off and replaced with fresh culture medium but addition of 100 nM Triptorelin did not affect levels of phosphorylated ERK1/2 Re-phosphorylation of ERK1/2 was less marked in HEK293_[SCL60] _cells and addition of Triptorelin considerably augmented levels of phosphorylated ERK1/2.

Levels of phosphorylated ERK1/2 (p-ERK1/2) in transfected MCF-7 cell clones were transiently elevated by GnRH receptor activation provided cells were incubated in serum-free medium overnight prior to stimulation. In the presence of serum, GnRH receptor activation did not significantly affect levels of p-ERK1/2 (Figure 5). Levels of p-ERK1/2 were not altered by GnRH receptor activation in serum-starved MDA-MB231-34 cells (Figure 5A).

Treatment of MCF-7hygro14 cells with 15-20 μM IGF-IR inhibitor II caused a rapid (within 30 minutes) and permanent decrease in levels of p-ERK1/2 in the presence of serum. The inhibitor did not elicit this effect in MDA-MB-231-34 cells (Figure 5C). When the inhibitor was washed off MCF-7hygro14 cells after a 1 h exposure followed by addition of medium containing serum, there was a rapid hyper-phosphorylation of ERK1/2 followed by a slow decline. Addition of 100 nM Triptorelin at the time of inhibitor wash-off did not significantly alter the intensity or dynamics of ERK1/2 phosphorylation (Figure 5D). The effects of IGFR-IR inhibitor II on p-ERK1/2 levels were similar in HEK293_[SCL60] _cells, with the exception that rapid hyper-phosphorylation of ERK1/2 did not occur when inhibitor was washed off unless Triptorelin was added (Figure 5D).

## Discussion

In this study, GnRH receptor immunostaining was found to be expressed over a wide dynamic range in breast cancer cases and its expression was significantly higher in patients with triple-negative disease, consistent with previous data [5,7]. High levels of expression were also observed in subgroups of luminal and HER2 breast cancers.

To investigate GnRH receptor function in breast cells, an immortalized human breast epithelial cell line (SVCT) and four well defined human breast cancer cell lines (MCF-7, MDA-MB-231, ZR-75-1 and T47D) were examined. None of the native cell lines possessed functional cell surface GnRH receptor detectable by binding assay or by induction of inositol phosphate production. Cell clones expressing high levels of GnRH receptor compared to other model systems could be isolated following transfection with GnRH receptor cDNA. In selected clones, treatment with GnRH agonist elicited high levels of inositol phosphate production, indicating that the receptor was functionally intact.

Despite the expression of high levels of GnRH receptor in SVCT-2, MCF-7hygro14 and MDA-MB-231-4, their growth was only marginally inhibited (SVCT-2) or was unaffected by treatment with the GnRH super-agonist Triptorelin in contrast to other model systems. By contrast, the growth of all cells was sensitive to IGF-IR or EGFR inhibitors (Figure 4). Analyses of receptor signaling indicated that Triptorelin significantly affected levels of phosphorylated ERK1/2 (p-ERK1/2) only in serum-starved transfected MCF-7 cells and GnRH receptor activation was unable to impinge on levels of p-ERK1/2 in MDA-MB-231-34 cells (Figure 5). In contrast, transient alterations in the levels of p-ERK1/2 do occur in cells which are growth-inhibited by GnRH receptor activation, even in the presence of growth factors (HEK293_[SCL60] _B35-2 neuroblastoma and prostate WPE-1-NB26-3) [8,9].

The lack of effect of GnRH agonist treatment on the growth of breast cell lines, and its limited effect on p-ERK1/2, may be explained by features of the growth-associated intracellular signaling apparatus within each breast cell line [31-39].

Growth of SVCT-2 cells was inhibited by IGF-IR inhibitor II, an inhibitor of ligand-induced IGF receptor auto-phosphorylation. Combined treatment with Triptorelin increased growth inhibition marginally (Figure 4). Thus the IGF-I signaling pathway is a candidate which may block anti-proliferative signaling by GnRH agonists in SVCT-2, consistent with transformation by SV40 [31,32].

Growth of MCF-7hygro14 was inhibited with IGF-IR inhibitor (Figure 4, IC_50 _was ~17 μM for these cells), consistent with the established growth-stimulatory effects of IGF-I in MCF-7 cells [33-36]. Furthermore, significant growth-inhibition over 4 days could be elicited by a brief exposure to IGF-IR inhibitor (2 hours). In MCF-7hygro14, the IGF-IR inhibitor caused a rapid decrease in the levels of p-ERK1/2, within 30 minutes (Figure 5) but it did not affect levels of p-ERK1/2 in MDA-MB-231-34 cells despite inhibiting their growth also. This is consistent with differences in signaling between the two cell lines [38] and the occurrence of mutationally activated k-Ras and B-Raf in MDA-MB-231-34 cells [37].

When IGF-IR inhibitor was washed off MCF-7hygro14 cells there was a rapid hyper-phosphorylation of ERK1/2, followed by a slow decline to basal levels, which was not influenced by GnRH receptor activation. Growth factors in the medium probably stimulate resurgence in ERK phosphorylation.

In comparison to MCF-7hygro14 cells, growth of HEK293_[SCL60] _cells was also inhibited by IGF-IR inhibitor but levels of p-ERK1/2 were relatively low in these cells compared to the breast cancer cells. Furthermore, hyper-phosphorylation of ERK1/2 did not occur in HEK293_[SCL60] _cells following removal of IGF-IR inhibitor. However, activation of GnRH receptor with Triptorelin following IGF-IR inhibitor wash-off did intensely elevate p-ERK1/2 levels (Figure 5). Intense transient activation of ERK-1/2 correlates with cell growth inhibition in HEK293_[SCL60] _cells [8,9]. This may not be the case in MCF-7 cells.

Perhaps these differences in the modulation of p-ERK 1/2 levels indicate that the IGF-IR-Ras-PI3K complex (which rapidly reforms when the IGF-IR inhibitor is washed off) is much more active in MCF-7 cells than in HEK293 cells. In MDA-MB231-34 cells, the activating c-Kirsten Ras and B-Raf mutations may be important for maintaining p-ERK1/2 levels independent of the effects of IGF-IR inhibitor on cell growth [37-39].

Estrogen receptor α influences IGF-IR, EGFR, Akt and MAPK activity by recruiting PI3K and Src to a microtubule-based protein scaffold [40]. Although ERα is present in MCF-7 cells and estrogen promotes MCF-7 growth, it is not endogenously expressed in MDA-MB-231 or HEK293 cells [40]. Hence, ERα may influence the signaling response to GnRH in MCF-7hygro14 relative to the other cells.

Differential signaling responses in MCF-7 and MDA-MB-231 cells (Figure 5) may reflect, at least in part, the activating mutations in PI3KCA and c-Kirsten Ras respectively [37,38] which impact upon MAPK-ERK1/2 activity. Other features of MDA-MB-231 cells [39] may contribute to the elevated basal phospholipase C activity in MDA-MB-231-34 (Figure 2C), where altered PKC activity may affect MAPK-ERK1/2 status in these cells.

Downstream from receptor-proximal interactions involving PI3K, Akt and PKC compete at the level of Raf-1 to exert opposite effects on the MAPK pathway (inhibitory and stimulatory, respectively) [41-44]. Perhaps constitutive activation of PI3K in MCF-7 cells abolishes the ability of GnRH-mediated PKC activation to impact upon Raf-1 in MCF-7-hygro14 cells. Interestingly, PKCα-mediated inhibition of Akt activity has been proposed as a mechanism for GnRH-mediated growth-inhibition in a mouse pituitary gonadotrope cell line immortalized with Sv40 T antigen [10].

Understanding how activating mutations in c-Kirsten Ras and B-Raf in MDA-MB-231 cells impact on GnRH receptor signaling to the MAPK cascade requires further investigation. In the presence of serum, levels of p-ERK1/2 are influenced by integration of signaling from multiple cell surface receptors (including FGF receptor in MDA-MB-231) [36], and this combined signaling probably prevents GnRH-mediated cell growth inhibition. The lack of effect of PI3K inhibitor on MCF-7hygro14 cell growth (Figure 4) suggests that simultaneous inhibition of both Akt and Ras signaling may be required to inhibit the growth of GnRH receptor positive cells [45,46].

## Conclusions

We discovered that GnRH receptor protein expression is often associated with triple negative breast cancer; however functional cell surface GnRH receptor levels are rare in cultured breast cell lines. The demonstration that a GnRH analog is ineffective in inhibiting growth of breast cancer cell lines expressing high levels of the GnRH receptor, despite eliciting robust signalling, provides a valuable tool for determining the intracellular context which does (eg HEK 293 and WPE-1-NB26-8) or does not (breast cancer cell lines) facilitate anti-proliferative effects of GnRH signalling. Creation and study of GnRH receptor positive models indicated that mitogenic signaling sensitive to IGF-IR inhibitor outweighs the potential growth-inhibitory effects of GnRH receptor activation in stably transfected breast cell lines. These results suggest that combinatorial strategies with growth factor inhibitors will be needed to enhance GnRH anti-proliferative effects in breast cancer.

## Abbreviations

GnRH: gonadotropin releasing hormone; IGF-I: insulin-like growth factor 1; EGF: epidermal growth factor; IGF-IR: insulin-like growth factor 1 receptor; IGF-IR inhibitor II: [N-(2-Methoxy-5-chlorophenyl)-N'-(2-methylquinolin-4-yl)-urea]; EGFR: epidermal growth factor receptor; EGFR/ErbB2 inhibitor: 4-(4-Benzyloxyanilino)-6,7-dimethoxyquinazoline]; ERα: estrogen receptor alpha; PI3K: phosphatidylinositol-4,5-bisphosphate 3-kinase; PI3KCA: PI3K catalytic subunit, PI3Kγ inhibitor- (5-Quinoxalin-6-ylmethylene-thiazolidine-2,4-dione); MEK, MAPK: mitogen activated protein kinase; ERK: extracellular signal regulated kinase; RACK: receptor for activated C- kinase.

## Competing interests

The authors declare that they have no competing interests.

## Authors' contributions

All authors read and approved the final manuscript. KM performed experiments, analyzed data and wrote the manuscript, CM, NM, IC and DF performed experiments and analyzed data, AHS and SPL assisted manuscript production and data interpretation, DJH and RPM contributed intellectually.

## Pre-publication history

The pre-publication history for this paper can be accessed here:

http://www.biomedcentral.com/1471-2407/11/476/prepub
